# Myasthenia Gravis Induced by Nivolumab: A Case Report

**DOI:** 10.7759/cureus.1702

**Published:** 2017-09-20

**Authors:** Jeet J Mehta, Eamon Maloney, Sachin Srinivasan, Patrick Seitz, Michael Cannon

**Affiliations:** 1 Internal Medicine/pediatrics, University of Kansas School of Medicine - Wichita; 2 Internal Medicine, University of Kansas School of Medicine - Wichita; 3 Cancer Center of Kansas, University of Kansas School of Medicine - Wichita

**Keywords:** nivolumab, myasthenia gravis, pd-1, immunotherapy, pd-1 inhibitor, monoclonal antibody, renal cell carcinoma, metastatic melanoma, ipilimumab

## Abstract

Nivolumab is a programmed cell death receptor (PD-1) inhibitor therapy for aggressive cancers; however, it poses a risk of immune-related adverse side effects. We present a 73-year-old male with renal cell carcinoma who developed myasthenia gravis (MG) after being treated with nivolumab, proven by acetylcholine receptor antibodies. Our patient presented with symptoms of fatigue and upper and lower extremity weakness, eventually resulting in respiratory failure as a result of MG. Nivolumab is an emerging therapy for advanced cancers but poses severe immune-related adverse events. Clinicians using PD-1 inhibitors should have a high index of suspicion of autoimmune diseases so that early discontinuation and treatment can be established to limit long-term morbidity and mortality.

## Introduction

Nivolumab, an immunoglobulin G4 monoclonal antibody, is a promising new immunotherapy for many cancers such as metastatic melanoma, non-small cell lung cancer, and renal cell carcinoma [1]. It works as a checkpoint inhibitor by binding to programmed cell death (PD-1) receptor to block programmed death ligand-1 (PD-L1) and programmed death ligand-2 (PD-L2) from binding T-cells. In addition to activating the immune system to target tumors, it also poses a risk of development of diseases, such as autoimmune thyroiditis, sarcoidosis, endophthalmitis, myasthenia gravis, and immune-related diabetes mellitus [1]. Myasthenia gravis, in particular, is a potential immune-related adverse effect that can develop in patients on other immunotherapies, such as pegylated-interferon and ipilimumab [2-3]. A recent case report identified myasthenia gravis in a patient with melanoma [4]. We present an elderly male with metastatic renal cell carcinoma (RCC) who developed myasthenia gravis after starting treatment with nivolumab. 

## Case presentation

A 73-year-old male with metastatic RCC presented with a four-day history of fatigue, hematuria, and progressive weakness in his upper and lower extremities. He had undergone a nephrectomy four years prior and temsirolimus therapy 12 weeks prior to hospitalization. Due to ineffective response, he was started on nivolumab two weeks before his presenting complaints. Four days after the second dose of nivolumab, he reported increased weakness, pain in his upper and lower extremities, and difficulty breathing. He required intubation due to poor respiratory effort and increased hypoxemia. He failed spontaneous breathing trials multiple times and required tracheostomy placement due to a prolonged course of intubation. Hospital stay was complicated by the development of pleural effusions, cardiac arrest, and Clostridium difficile infection.

**Figure 1:**
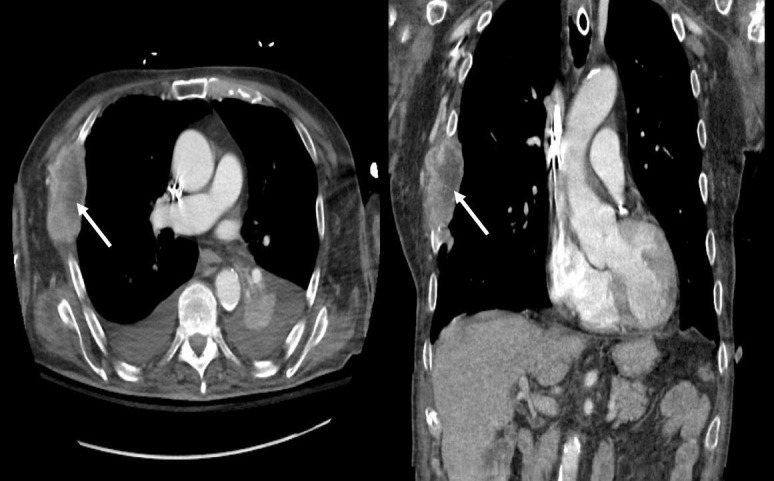
Computed Tomography of the Chest with Lytic Lesion on Right Ribs

Labs were consistent with rhabdomyolysis with a creatinine phosphokinase (CPK) of 8,950 U/L, an elevated serum aspartate aminotransferase (AST) of 1,066 U/L, and an elevated alanine aminotransferase (ALT) of 824 U/L. Muscle and nerve biopsies showed no definitive pathology. Cerebrospinal fluid studies, including cell counts with differential and cultures for viral and bacterial organisms, were negative. Electromyography showed denervation potential in all tested muscles. An acute hepatitis panel was negative. The patient’s acetylcholine receptor (AChR) antibody returned positive (8.70 nmol/L). Computed tomography of the chest was unremarkable for thymoma but was remarkable for a metastatic lytic lesion of his right ribs as can be seen in Figure 1. The patient likely had an underlying paraneoplastic myasthenia gravis unmasked by nivolumab. He was initially treated with steroids and pyridostigmine (first dose of 30 mg, then escalated to 120 mg every four hours). Due to minimal improvement with the aforementioned initial treatments, the patient underwent five courses of plasmapheresis and subsequent intravenous immunoglobulin (IVIG) therapy. CPK and transaminase levels trended down after treatment of different modalities; however, it was difficult to determine his level of responsiveness to therapy as there was a component of disuse atrophy. He was eventually transferred to a long-term acute care facility. His prognosis remained poor, given his metastatic renal cell carcinoma and chronic respiratory failure. He was discharged with pyridostigmine, 60 mg q4h while awake, and a steroid taper. 

## Discussion

Myasthenia gravis is a debilitating disease with a prevalence of 20 per 100,000 in the United States population [5]. It presents with symptoms of fatigue, diplopia, respiratory insufficiency, and distal extremity weakness. The diagnosis of myasthenia gravis is confirmed with AChR antibodies which have a sensitivity of up to 90% [6]. Forty percent of patients who are negative for AChR will be positive for muscle receptor tyrosine kinase (MuSK) antibodies [7]. Treatment consists of pyridostigmine for acute symptoms. Limited evidence from randomized control trials suggests glucocorticoids can provide benefit chronically [8]. Plasmapheresis suggests short-term benefits in case-control trials [9]. IVIG provided a clinical improvement in moderate to severe myasthenia gravis compared to placebo in one clinical trial [10]. The evidence, however, is insufficient to compare its efficacy to plasmapheresis at this time. Our patient was treated with all modalities with little improvement. In addition to myasthenia gravis, rhabdomyolysis and transaminitis are other potential adverse effects of nivolumab. Myasthenia gravis and other autoimmune diseases can be seen with immunotherapy with nivolumab alone. Treatment of nivolumab-induced myasthenia gravis can be complicated by other comorbidities, such as prolonged immobility and metastatic cancer.

## Conclusions

Nivolumab is an emerging therapy for advanced cancers; however, the occurrence of immune-related adverse events is a significant risk factor. The mechanism of myasthenia gravis secondary to PD-1 inhibitor treatment is unclear and further post-marketing surveillance data are needed to establish true incidence. Clinicians using PD-1 inhibitors should have a high index of suspicion of myasthenia gravis so that early discontinuation and treatment can be instituted to limit long-term morbidity and mortality. 
